# A Multiple Correspondence Analysis of Patterns of CBD Use in Hemp and Marijuana Users

**DOI:** 10.3389/fpsyt.2020.624012

**Published:** 2021-01-14

**Authors:** Joseph R. Vilches, Mackenzie B. Taylor, Francesca M. Filbey

**Affiliations:** ^1^Center for BrainHealth, School of Behavioral and Brain Sciences, University of Texas at Dallas, Dallas, TX, United States; ^2^Department of Psychology, University of North Texas, Denton, TX, United States

**Keywords:** cannabidiol, marijuana, multiple correspondance analysis, cbd, thc, HEMP

## Abstract

**Background:** With the passing of the 2018 Agriculture Improvement Act that legalized hemp-derived products, i.e., cannabidiol (CBD), the use of CBD has increased exponentially. To date, the few studies that have characterized individuals who use CBD suggest that co-use of CBD and tetrahydrocannabinol (THC)-dominant cannabis, i.e., marijuana, is highly prevalent. It is, therefore, important to investigate the relationship between CBD use and marijuana use to understand the antecedents and consequences of co-use of these two cannabis products.

**Methods:** We conducted an online survey using structured questionnaires to determine differences in CBD users with (CBD+MJ) and without co-morbid marijuana use. Group comparisons were carried out using chi-square tests and ANOVA. Multiple correspondence analysis (MCA) with bootstrap ratio testing was performed to examine the relationship between the categorical data.

**Results:** We received 182 survey responses from current CBD users. CBD+MJ had more types of CBD administration (*F* = 17.07, *p* < 0.001) and longer lifetime duration of CBD use (χ2 = 12.85, *p* < 0.05). Results from the MCA yielded two statistically significant dimensions that accounted for 77% of the total variance. Dimension 1 (representing 57% of the variance) associated CBD+MJ with indication of CBD use for medical ailments, use of CBD for more than once a day for longer than 2 years, applying CBD topically or consuming it via vaping or edibles, being female, and, having lower educational attainment. Dimension 2 (representing 20% of the variance) separated the groups primarily on smoking-related behaviors where CBD+MJ was associated with smoking CBD and nicotine.

**Conclusions:** Identifying the factors that influence use of CBD and marijuana can inform future studies on the risks and benefits associated with each substance as well as the impacts of policies related to cannabis-based products.

## Introduction

The *cannabis sativa* plant species contains a multitude of varieties, such as hemp and marijuana (MJ), with various active elements known as phyto-cannabinoids. Hemp and MJ are commonly differentiated according to their concentration levels of delta-9 tetrahydrocannabinol (THC), the main psychoactive phyto-cannabinoid found in *cannabis sativa*. Hemp is classified as *cannabis sativa* with a THC concentration lower than 0.03%, while those with a concentration >0.03% are classified as MJ (1, 2). Prior to 2018, both hemp and MJ were classified as schedule I substances. In December of 2018, the United States Senate passed The Agriculture Improvement Act. Under this new law, hemp was rescheduled from a DEA schedule I substance to a schedule V substance. This reclassification identifies hemp and hemp-derived products, such as cannabidiol (CBD), as a substance of medicinal value with no addictive properties and legalizes it nationally. CBD's appeal as a medicinal agent is based upon its favorable tolerance in both human and animal models (3–5). These models found a lack of habit-forming potential (6, 7) and rare incidents of adverse side-effects (8) from CBD use compared to THC (9–11).

To date, cannabis research has focused largely on THC and CBD given that they are the main phyto-cannabinoids found in *cannabis sativa* (7, 10, 12)]. In isolation, THC has been shown to induce psychoactive and appetitive effects (13) and impact cognitive abilities, including but not limited to attention, and episodic memory (14, 15). CBD, on the other hand, has been shown to have anxiolytic (16), antipsychotic (17), and neuroprotective effects (18–21).

Studies have found high co-use of THC and CBD, i.e., >50% in CBD users (22, 23) that highlight the need to understand how the two substances interact. To date, however, our knowledge of this interaction remains largely inconclusive. While it has been suggested that CBD does not impact THC's subjective and reinforcing properties (24), its modulatory role on THC's effects on cognition is mixed. For example, some studies have found that CBD has a protective effect on THC-related episodic memory deficits (25), such that cannabis users who smoked cannabis high in cannabidiol content showed no memory impairment. On the other hand, CBD was not found to modulate THC's effect on attention (26, 27). Timing of administration and THC/CBD ratio further complicates this interaction (28). For example, when CBD is administered prior to THC it has been shown to potentiate its effects, but this potentiation does not occur when they are administered concurrently (28).

Thus, there is a critical gap in the knowledge surrounding co-use of CBD and THC. This paucity in the literature combined with the increasing prevalence of both CBD and MJ use, highlight the importance of examining simultaneous use of CBD and MJ. The purpose of the present study was to investigate multivariate patterns that are associated with isolated use vs. co-use of MJ and CBD.

## Methods

The present cross-sectional survey study was conducted with Internal Review Board approval from the University of Texas at Dallas.

### Respondents

We recruited adults who self-reported CBD use via online advertisements posted on Dallas-Fort Worth and CBD forums (Reddit, Craigslist, Discord, and NextDoor). Inclusion criteria for all respondents was as follows: the endorsement of current CBD use, aged 18 years or older, and, written informed consent.

The study was conducted online in its entirety via Qualtrics Research Software (29). Respondents from the advertisements were directed to the web-based survey in order to participate in the study. The first page of the survey described the informed consent procedures. In order to ensure understanding of the purpose and procedure of the study, the respondents were asked to answer three multiple choice questions about the study. Answering all of the questions correctly was a pre-requisite for informed consent. Those who answered all of the questions correctly were then asked to provide a digital signature to document informed consent to participate in the study. After the digital signature page was completed, the survey assessments began. Those who did not provide a digital signature could not progress with the survey assessments. No identifying information was collected in this survey.

Compensation for study participation was optional. Those who opted for compensation were directed to a different survey. This kept the “data collection” survey and “optional compensation” survey separate such that information could not be linked to respondents' identifying information, thus ensuring anonymity. Following compensation, information from the “optional compensation” survey was destroyed.

### Assessments

The survey used in the present study was adapted from Corroon and Phillips (22) and was created using Qualtrics survey software (29). This survey included questions designed to measure respondent history of use, rate of use, method of self-administration, and the medical indication of CBD use. We also collected sociodemographic data including biological sex, age, and highest level of education. In order to measure respondents' cannabis, nicotine, and alcohol use behavior the following assessments were included in the survey: the Cannabis Use Disorders Identification Test—Revised [CUDIT-R (30)], The Fagerstrom Test for Nicotine Dependence—Revised, [FTND-R; (31)], and the Alcohol Use Disorder Identification Test [AUDIT; (32)]. Quality control of participant responses was carried out using recommendations from Teitcher et al. (33) that examined response times as a metric to detect outliers and examining response patterns to detect dubious responses.

### Data Analyses

All analyses were conducted in RStudio (34) using R 3.6.3 (35). Descriptive statistics were calculated to examine CBD use characteristics, sociodemographic variables, methods of CBD administration, medicinal CBD use, cannabis, nicotine, and alcohol use characteristics. Chi-square and ANOVA tests were used for comparisons of MJ endorsement groups across variables.

**Figure 1:**
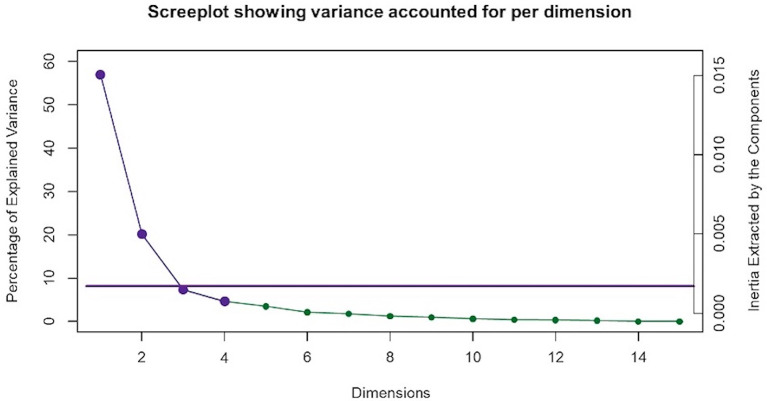
Multiple correspondence analysis (MCA) screeplot. MCA identified four significant dimensions accounting for a combined total of 89% of the variance. The screeplot shows two statistically significant dimensions (dimensions 1 and 2) that survived the Kaiser line test and accounted for 77% of the variance. The purple Kaiser line is a visual representation of the “screetest.”

To elucidate possible relationships between multiple variables, multiple correspondence analysis (MCA) in the ExPosition package (36) was used. MCA is an extension of correspondence analysis (CA) and a generalization of principal component analysis (PCA). It is a multivariate analysis technique that allows for the investigation of potential relationships between multiple categorical variables (37–40). Similar to PCA, MCA dimensions are orthogonal to each other and independently explain as much of the variance as possible (41, 42). The Kaiser line test was performed to determine the number of dimensions to retain for further analysis. This test is based on the Kaiser criterion, which recommends retention of dimensions with eigenvalues ≥1. The purple points in Figure 1, show dimensions with eigenvalues that meet Kaiser criterion. The line is generated based on the relative location of the “elbow” of the scree plot where the variance represented by one dimension is not statistically different than that of the next (43). MCA reduces the number of dimensions seen in a given dataset and converts both variables and respondents into factor scores. This factor score calculation and data dimension reduction allow for the visual representation of both variables and respondents along a two-dimensional plane. When examining the factor plots, points (representing variables or respondents) that are plotted closer together have a greater association with each other (38, 39, 44, 45). Variable stability and statistical inferences pertaining to MJ group differences were evaluated via bootstrap resampling (46), bootstrap ratio and 95% confidence interval calculation (47), all of which were carried out with the InPosition package (36). The significance threshold for all analyses was set at *p* < 0.05.

## Results

### Participant Characteristics

Two hundred and forty-five individuals responded to the online survey. Of these, 53 had partial data and were excluded. Of the complete surveys, nine had response times classified as outliers using the graphics package from R 3.6.3 (35) and were consequently excluded. Lastly, one respondent's response pattern exhibited signs of malingering and was also excluded. In this study, malingering was defined as having the same response (e.g., all “yes” or “10”) to all of the survey questions that also then conflict with each other. In this particular case, the respondent endorsed the most extreme answer in the Likert scale questions and answered “yes” for every yes or no binary question. This pattern revealed inconsistent responses across similar questions. After these quality control steps, a total of 182 respondents were included in further analyses (112 males, 70 females). See Table 1 for respondents' demographic and drug use information. Respondents were classified into concurrent MJ and CBD use (CBD + MJ) (*N* = 105), and, CBD only use (*N* = 77). The two groups were significantly different in age [χ^2^(5) = 15.67, *p* = 0.008], education [χ^2^(7) = 15.30, *p* = 0.032], and nicotine use [χ^2^(1) = 15.67, *p* = 0.007]. CBD+MJ users were younger, had less years of education and greater nicotine use than CBD only users. CBD+MJ users reported greater number of CBD self-administration methods [*F*_(1,180)_ = 16.73, *p* < 0.001, $\eta_{p}^{2}$ = 0.09]. Specifically, there were significant differences between CBD+MJ users and CBD only users in the following CBD self-administration methods: sublingual [χ^2^(1) = 4.45, *p* = 0.035, vaping χ^2^(1) = 6.07, *p* = 0.014], smoking [χ^2^(1) = 21.49, *p* = 0.001] and edible [χ^2^(1) = 5.39, *p* = 0.020] administration (Table 2).

**Table 1:** Respondents' demographic information.

	**Total all**	**CBD+MJ**	**CBD only**	***p-*value**
	**(*N* = 182)**	**(*N* = 105)**	**(*N* = 77)**	
**Biological Sex**				0.669^a^
Male	112 (61.5%)	66 (62.9%)	46 (59.7%)	
Female	70 (38.5%)	39 (37.1%)	31 (40.3%)	
**Age group**				0.008^a^
18–24	53 (29.1%)	41 (39.0%)	12 (15.6%)	
25–34	74 (40.7%)	42 (40.0%)	32 (41.6%)	
35–44	34 (18.7%)	14 (13.3%)	20 (26.0%)	
45–54	16 (8.8%)	6 (5.7%)	10 (13.0%)	
55–64	3 (1.6%)	1 (1.0%)	2 (2.6%)	
65 or Over	2 (1.1%)	1 (1.0%)	1 (1.3%)	
**Education**				0.032^a^
No high school	3 (1.6%)	3 (2.9%)	0 (0.0%)	
High school/GED	17 (9.3%)	14 (13.3%)	3 (3.9%)	
Some college	60 (33.0%)	35 (33.3%)	25 (32.5%)	
Associate degree	19 (10.4%)	14 (13.3%)	5 (6.5%)	
Bachelor's degree	58 (31.9%)	28 (26.7%)	30 (39.0%)	
Master's degree	20 (11.0%)	8 (7.6%)	12 (15.6%)	
Doctoral degree	3 (1.6%)	1 (1.0%)	2 (2.6%)	
Professional	2 (1.1%)	2 (1.9%)	0 (0.0%)	

a *Pearson's Chi-squared test*.

*CBD, cannabidiol; MJ, marijuana; CBD+MJ, respondents with CBD and marijuana use*.

**Table 2:** CBD use in the study sample.

**CBD use measure**	**All**	**MJ+CBD**	**CBD only**	***p*-value**
	**(*N* = 182)**	**(*N* = 105)**	**(*N* = 77)**	
**How often do you use CBD?**				0.243^a^
Less than once a day	77 (42.3%)	48 (45.7%)	29 (37.7%)	
Daily	84 (46.2%)	43 (41.0%)	41 (53.2%)	
More than once a day	21 (11.5%)	14 (13.3%)	7 (9.1%)	
**CBD use history**				0.065^a^
Less than one month	11 (6.0%)	4 (3.8%)	7 (9.1%)	
Less than three months	13 (7.1%)	6 (5.7%)	7 (9.1%)	
<6 months	44 (24.2%)	25 (23.8%)	19 (24.7%)	
<1year	39 (21.4%)	22 (21.0%)	17 (22.1%)	
1–2 years	53 (29.1%)	29 (27.6%)	24 (31.2%)	
More than 2 years	22 (12.1%)	19 (18.1%)	3 (3.9%)	
**Sublingual administration**				0.023^a^
No	134 (73.6%)	84 (80.0%)	50 (64.9%)	
Yes	48 (26.4%)	21 (20.0%)	27 (35.1%)	
**Vaping Administration**				0.009^a^
No	125 (68.7%)	64 (61.0%)	61 (79.2%)	
Yes	57 (31.3%)	41 (39.0%)	16 (20.8%)	
**Capsule administration**				0.985^a^
No	163 (89.6%)	94 (89.5%)	69 (89.6%)	
Yes	19 (10.4%)	11 (10.5%)	8 (10.4%)	
**Liquid administration**				0.656^a^
No	151 (83.0%)	86 (81.9%)	65 (84.4%)	
Yes	31 (17.0%)	19 (18.1%)	12 (15.6%)	
**Smoking administration**				<0.001^a^
No	123 (67.6%)	56 (53.3%)	67 (87.0%)	
Yes	59 (32.4%)	49 (46.7%)	10 (13.0%)	
**Edible administration**				0.013^a^
No	121 (66.5%)	62 (59.0%)	59 (76.6%)	
Yes	61 (33.5%)	43 (41.0%)	18 (23.4%)	
**Topical administration**				0.518^a^
No	135 (74.2%)	76 (72.4%)	59 (76.6%)	
Yes	47 (25.8%)	29 (27.6%)	18 (23.4%)	
**Number of CBD use methods**	1.77 (1.04)	2.03 (1.17)	1.42 (0.69)	<0.001^b^
**FTND scored**	0.64 (1.80)	0.88 (2.09)	0.31 (1.24)	0.036^b^
**AUDIT scored**	5.89 (5.55)	7.01 (6.44)	4.36 (3.52)	0.001^b^
**CUDIT scored**	-	7.68 (5.17)	-	-

a *Pearson's Chi-squared test*.

b *Linear Model MANOVA*.

*CBD, cannabidiol; MJ, marijuana; CBD+MJ, respondents with CBD and marijuana use; FTND, the Fagerstrom Test for Nicotine Dependence—Revised; CUDIT, the Cannabis Use Disorders Identification Test—Revised; AUDIT, the Alcohol Use Disorder Identification Test*.

### Multiple Correspondence Analysis (MCA)

**Figure 2:**
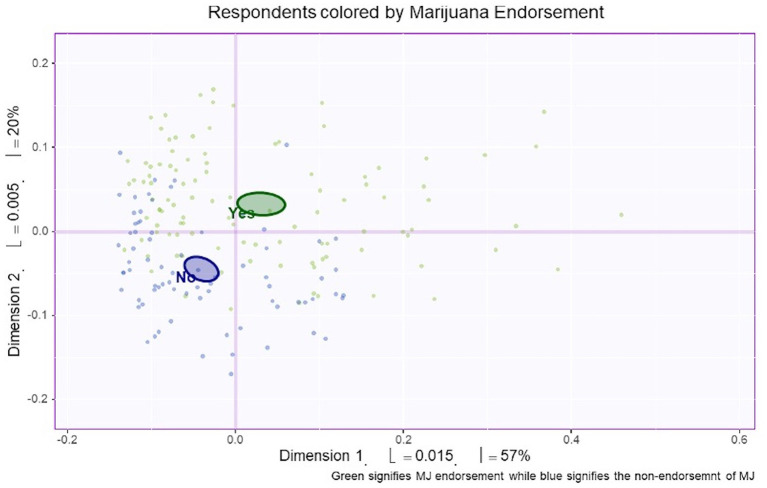
Bootstrap confidence interval comparisons between CBD users with and without marijuana co-use. Mean confidence intervals were created from the bootstrap resampling. Respondents were classified according to endorsement of marijuana use. Based on this figure, dimension 1 (the horizontal line) and dimension 2 (the vertical line) separated CBD users with (green) and without (purple) concurrent marijuana use.

**Figure 3:**
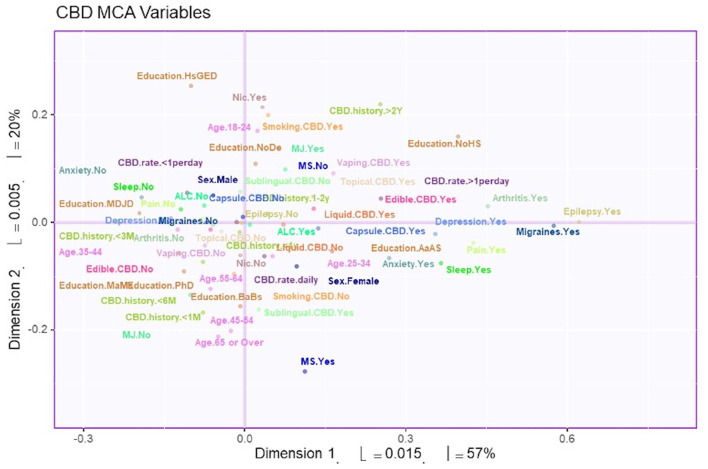
Survey variables plotted on dimensions 1 and 2. The variable factor scores plotted to show dimensions 1 and 2. These two dimensions account for 77% of the total variance. Distance from the axis indicates the association of the variable to the dimension. In addition, two points that are close to each other have greater association with each other.

MCA identified four significant dimensions accounting for a combined total of 89% of the variance (see Figure 1). Dimensions 1 and 2 survived the Kaiser line test and were retained for further analyses. Together these two dimensions accounted for 77% of the variance. Dimension 1 accounted for 57%, while dimension 2 accounted for 20% of the variance. 95% mean confidence intervals via bootstrap resampling showed that dimension 2 best separated CBD+MJ respondents from CBD only respondents (see Figure 2). Based on the variable factor score map (see Figure 3), dimension 1 separated respondents primarily based on ailments indicated for the use of CBD. CBD+MJ use was associated with endorsement of ailments (anxiety, depression, physical pain, arthritis, migraines, and sleep disorders), high school level of education, being female, administration of CBD via topical, edible, and vaping, and using CBD more than once a day for longer than 2 years (see top right quadrant of Figure 3 and Table 3). CBD only use was associated with absence of ailments related to CBD use, possession of advanced graduate degrees (i.e., master's degree), fewer types of CBD administration, and use of CBD less than once a day and <3 months (see lower left quadrant of Figure 2 and Table 3).

**Table 3:** Ailments attributed to CBD use.

**Disorder**	**Total**	**MJ+CBD**	**CBD only**	***p-*value**
	**(*N* = 182)**	**(*N* = 105)**	**(*N* = 77)**	
Anxiety	76 (41.8%)	48 (45.7%)	28 (36.4%)	0.206^a^
Depression	51 (28.0%)	35 (33.3%)	16 (20.8%)	0.062^a^
Pain	29 (15.9%)	19 (18.1%)	10 (13.0%)	0.352^a^
Arthritis	21 (11.5%)	16 (15.2%)	5 (6.5%)	0.068^a^
Migraines	20 (11.0%)	14 (13.3%)	6 (7.8%)	0.238^a^
Sleep disorders	45 (24.7%)	32 (30.5%)	13 (16.9%)	0.036^a^
Epilepsy	2 (1.1%)	1 (1.0%)	1 (1.3%)	0.825^a^
Multiple sclerosis	7 (3.8%)	2 (1.9%)	5 (6.5%)	0.112^a^

a *Pearson's Chi-squared test*.

*CBD, cannabidiol; MJ, marijuana; CBD+MJ, respondents with CBD and marijuana use*.

**Figure 4:**
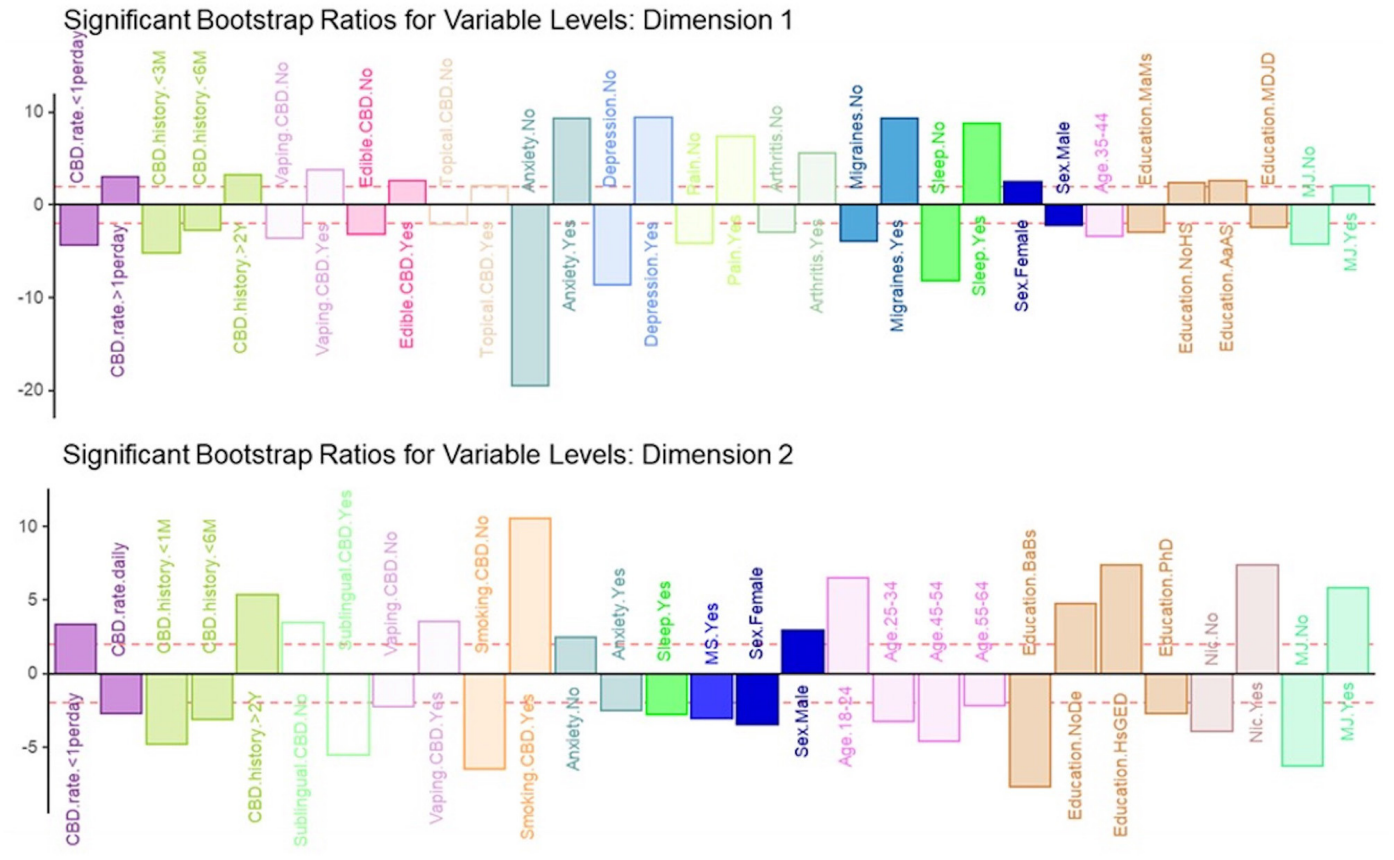
Significant bootstrap ratios for dimensions 1 & 2. Illustration of the significant bootstrap ratios for the variables for dimensions 1 and 2. Bars that are filled-in represent variables with a bootstrap ratio >2. Variables in the same side of the y-axis are positively associated with each other.

Dimension 2 primarily separated respondents based on CBD and nicotine smoking behaviors. CBD+MJ use was associated with smoking and vaping CBD, use of CBD for more than 2 years at a rate of less than once day, smoking nicotine, <2 years of college level education, being male and between the ages of 18–24. CBD only use was associated with using CBD sublingually daily for <6 months, possession of a college education, being between the ages of 25–64, and self-reported anxiety, sleep disorders, MS (Figure 4).

## Discussion

The present study sought to elucidate the factors that contribute to co-use of CBD and MJ. MCA was used to explore multivariate relationships within the data, yielding two MCA dimensions, which accounted for the majority of variance. Dimension one separated the CBD only users from CBD+MJ users primarily on ailments for which CBD was used for—anxiety, depression, physical pain, arthritis, migraines, and sleep disturbances. Dimension two separated the groups based on smoking CBD and nicotine.

### MCA Dimension 1

Our results suggest that co-use of MJ in CBD users is associated with indication of CBD use for medical ailments, use of CBD for more than once a day for longer than 2 years, applying CBD topically or consuming it via vaping or edibles, being female, and having lower educational attainment. Regarding the medical ailments found in MCA dimension 1—anxiety, depression, physical pain, arthritis, migraines, and sleep disorders -, we found that the presence of one ailment was associated with the possible presence of other ailments. Given high co-morbidity between psychiatric disorders, it isn't surprising that anxiety and depression were associated in CBD+MJ users. For example, anxiety has been linked with both depression and substance use (48, 49) and is particularly prevalent in marijuana users (50, 51). Although CBD is more widely considered to provide relief from symptoms related to pain, arthritis and sleep disturbances, we found that MJ use in CBD users was associated with presence of these conditions. It is possible that these individuals either experience or have expectancies that MJ use in addition to CBD provides greater relief for these ailments. There is existing literature that describes the “entourage effect” in cannabis where full spectrum cannabis products that maintain the full profile of the cannabis plant leads to increased endogenous cannabinoid levels that are above and beyond that of the individual phytocannabinoid's isolated components, making them more efficacious for a variety of medical ailments (52). Indeed, medical MJ that contain a variety of cannabinoids including THC, CBD, as well as other cannabinoids and terpenes is often indicated for relief of epilepsy, movement disorders, and pain (53–55). In pain studies, 1:1 THC:CBD (Sativex) combinations have been shown to be more efficacious for cancer-related, arthritis, and other chronic pain compared to both placebo and THC isolate (56–58). In studies involving MS patients, THC (2.7 mg Tetranabinex) and CBD (2.5 mg Nabidiolex) dominant medications were shown to produce pain relief, but a 1:1 THC:CBD combination drug (Sativex) significantly improved sleep symptoms and pain above the other two (59). These initial studies demonstrate that 1:1 THC:CBD combination drugs provide greater symptom relief than isolates in clinical populations. It is also possible that this association could be due to known associations of mood disorders with medical conditions such as chronic pain, arthritis, sleep disturbances (60–62) and may play a mediating role between pain and sleep disturbances in arthritis patients (61). In this instance, pain may contribute to exacerbated depression symptoms in the long-term which, in turn, can result in sleep disturbances. Given the large literature on the associations between marijuana use and mood disorders, we speculate that this may also explain why mood disorders and medical conditions were associated with CBD+MJ users.

Previous results demonstrating that the use of both MJ and CBD is associated with a need for pain relief are consistent with our findings, as the bootstrap ratios indicated that both physical pain and endorsement of MJ co-use were related. The underlying mechanisms for the analgesic effect of CBD are subject to debate. However, previous studies have proposed CBD's interaction with the glycine and serotonergic systems as possible vehicles (63). In animal models of arthritis, locally applied CBD has been found to lessen joint pain and inflammation (64–66). This finding may explain why the endorsement of administering CBD topically was associated with the indication of CBD use for ailments such as chronic pain and arthritis.

We also found that CBD+MJ users are more likely to be female, which is concordant with results showing that female MJ users were more likely to report MJ use for the treatment of pain compared to male MJ users (67). Previous studies have shown using CBD more than once a day is associated with medicinal use (22). The perceived medicinal benefits could be a contributing factor to high rates of CBD use, despite a likelihood of a deep overestimation about the efficacy of CBD has been demonstrated (22, 23). Nevertheless, the literature corroborates our finding that co-use of CBD and MJ is more related to co-existing medical ailments than CBD use alone.

### MCA Dimension 2

Our results for dimension 2 from the MCA suggest that being young (18–24 years old), male, having an associate degree or less, and the use of nicotine products is associated with the endorsement of MJ co-use. The findings are in accordance with previous research showing that 18–25-year-olds have the highest rate of MJ use (68), and that MJ users tend to have lower levels of education compared to non-users (69, 70). Previous studies have found that earlier initiation of MJ use was associated with lower academic and career attainment (71, 72), suggesting that CBD use may not mitigate the detrimental effects of MJ use.

Nicotine use was found to be a significant a variable associated with MJ co-use. The co-use of nicotine with MJ has been shown in previous research, with data suggesting that greater exposure to one, is associated with greater exposure to the other (73). When examining the CBD history variables, it was found that using CBD less than once a day for longer than 2 years was associated with the endorsement of MJ co-use. The sporadic use history of CBD seen in MJ users could be due to CBD exerting a non-effect on the subjective rewarding effects of THC (24). From the bootstrap ratios, smoking CBD seemed to have the highest association with the endorsement of MJ co-use. This finding makes sense pharmacologically speaking, as smoking has been found to yield the highest plasma concentration in the shortest amount time in both CBD (74) and MJ use (75, 76). In this instance, smoking and vaping methods of administration could be associated with MJ and CBD co-use due to increased familiarity with these methods in MJ users. This is in line with previous studies showing that both vaping and smoking are popular methods of administration in experienced MJ users (77, 78).

Previous findings have suggested that even though the effects of THC and CBD do not physiologically influence each other, the high rate of MJ co-use in the CBD using population may in part be due to MJ users having greater familiarity with CBD (22). The results of the present study support this claim as co-use was associated with using CBD longer but infrequently. Additionally, the methods of CBD administration that were associated with MJ use were methods that are most commonly seen in MJ use (e.g., edibles, vaping, and smoking) (79, 80).

## Conclusions and Limitations

Our findings suggest that co-use of MJ in CBD users may be influenced by several factors, with medical ailments and smoking behavior being primary factors. Although the co-use of MJ in CBD users is associated with factors that have been widely reported to be associated with MJ use, it is surprising to note that the presence of both psychological and medical conditions is more associated with CBD+MJ use than CBD use alone. This suggests that the use of these substances for symptom relief should be an important consideration for future studies.

### Limitations

Due to the cross-sectional nature of the present study, the temporal relationship between CBD use and MJ use cannot be established. The present study also relied on self-reported measures and must take into account issues with reliability. Several studies have explored the reliability and validity of survey measures, including those performed online via similar platforms such as those used in this study. These studies have found that respondents tend to use satisficing or choosing “good enough” answers which increases consistency, reliability, and convergent validity of measures but decreases discriminant validity (81). This, along with our quality control procedures and our use of previously validated questionnaires may mitigate some of the potential limitations of the survey approach. Furthermore, we followed recommendations from previous studies such as: designing the questionnaire in such a way to improve response rates, piloting the survey prior to distribution, and only asking questions that are applicable toward our research goal (82). Based on these recommendations and guidance provided by previous research on using survey approaches to measure substance use (83), we constructed our measurements and analytic approach to avoid common pitfalls. For example, in the survey we emphasized the confidentiality of all information provided by respondents and only used validated measures to minimize measurement error.

Additionally, there is no certainty that the survey respondents truly were diagnosed with the psychiatric conditions they endorsed. In this instance, we assume respondents are taking CBD for symptoms related to endorsed ailments, but these statements cannot be confirmed without professional diagnoses. Moreover, it is likely that due to the nature of the study respondents may have under-estimated their frequency of self-administration, tolerance, and other dependence symptoms.

## Data Availability Statement

The raw data supporting the conclusions of this article will be made available by the authors, without undue reservation.

## Ethics Statement

The studies involving human participants were reviewed and approved by Internal Review Board, University of Texas at Dallas. The patients/participants provided their written informed consent to participate in this study.

## Author Contributions

JV developed the study concept and design, conducted the acquisition, analyses and interpretation of the data, and drafted the manuscript. MT contributed to the data analyses and interpretation, and drafted the manuscript. FF contributed to the concept and design of the study and critical revisions and approval of the submitted manuscript. All authors contributed to the article and approved the submitted version.

## Conflict of Interest

The authors declare that the research was conducted in the absence of any commercial or financial relationships that could be construed as a potential conflict of interest.
